# Efficacy of Mechanical Thrombectomy using Penumbra ACE^TM^ Aspiration Catheter Compared to Stent Retriever Solitaire^TM^ FR in Patients with Acute Ischemic Stroke

**DOI:** 10.3390/brainsci11040504

**Published:** 2021-04-16

**Authors:** Dalibor Sila, Markus Lenski, Maria Vojtková, Mustafa Elgharbawy, František Charvát, Stefan Rath

**Affiliations:** 1 Department of Neurosurgery and Interventional Neuroradiology, Donau-Isar Klinikum, Perlasberger Str. 41, 94469 Deggendorf, Germany; stefan.rath@donau-isar-klinikum.de; 2 Neurosurgical Clinic, Campus Grosshadern, Clinic of the University of Munich (LMU), Marchioninistrasse 15, 81377 Munich, Germany; markus.lenski@med.uni-muenchen.de; 3 Department of Statistics, Faculty of Economic Informatics, University of Economics in Bratislava, Dolnozemska cesta 1/b, 85235 Bratislava, Slovakia; maria.vojtkova@euba.sk; 4 Department of Radiology and Interventional Radiology, Donau-Isar Klinikum, Perlasberger Str. 41, 94469 Deggendorf, Germany; mustafa.elgharbawy@donau-isar-klinikum.de; 5 Radiodiagnostic Departement, Military University Hospital Prague, U Vojenské nemocnice 1200, 16902 Praha, Czech Republic; frantisek.charvat@uvn.cz

**Keywords:** thrombectomy, stroke, stent retriever, aspiration catheter, procedure efficacy, solitaire, penumbra

## Abstract

Background: Mechanical thrombectomy is the standard therapy in patients with acute ischemic stroke (AIS). The primary aim of our study was to compare the procedural efficacy of the direct aspiration technique, using Penumbra ACE^TM^ aspiration catheter, and the stent retriever technique, with a Solitaire^TM^ FR stent. Secondarily, we investigated treatment-dependent and treatment-independent factors that predict a good clinical outcome. Methods: We analyzed our series of mechanical thrombectomies using a Solitaire^TM^ FR stent and a Penumbra ACE^TM^ catheter. The clinical and radiographic data of 76 patients were retrospectively reviewed. Using binary logistic regression, we looked for the predictors of a good clinical outcome. Results: In the Penumbra ACE^TM^ group we achieved significantly higher rates of complete vessel recanalization with lower device passage counts, shorter recanalization times, shorter procedure times and shorter fluoroscopy times (*p* < 0.001) compared to the Solitaire^TM^ FR group. We observed no significant difference in good clinical outcomes (52.4% vs. 56.4%, *p* = 0.756). Predictors of a good clinical outcome were lower initial NIHSS scores, pial arterial collateralization on admission head CT angiography scan, shorter recanalization times and device passage counts. Conclusions: The aspiration technique using Penumbra ACE^TM^ catheter is comparable to the stent retriever technique with Solitaire^TM^ FR regarding clinical outcomes.

## 1. Introduction

The goal for the treatment of patients with acute ischemic stroke (AIS) due to proximal cerebral vessel occlusion is a quick perfusion restoration of the involved territory to minimize brain tissue damage. Endovascular treatment with mechanical thrombectomy using second-generation devices, particularly stent retrievers, improves the clinical outcome without increased procedural complications compared to intravenous (IV) administration of recombinant tissue plasminogen activator (rtPA) [1,2,3,4,5,6,7]. A direct contact thrombus aspiration as a first pass technique (ADAPT) has comparable outcomes to the stent retriever technique [8,9], and shows better results compared to treatment by IV administration of rtPA alone, in relation to good clinical outcomes [10]. The primary aim of our study was a comparison of the procedural efficacy of the aspiration technique using Penumbra ACE^TM^ aspiration catheter and the stent retriever technique with a Solitaire^TM^ FR stent. Secondarily, we tried to find relevant treatment-dependent and treatment-independent predictive factors concerning a good clinical outcome.

## 2. Materials and Methods

We analyzed patients with AIS treated with intra-arterial mechanical thrombectomy at the Neurosurgical and Neurointerventional Department of Donau-Isar Klinikum in Deggendorf (Germany) from July 2012 to December 2017. Mechanical thrombectomy was performed by three senior hybrid neurosurgeons who had an average of ten years of endovascular experience. Each of them performs 20–30 mechanical thrombectomy interventions per year. The number of interventions increased each year and, since 2017, we have performed approximately 80 mechanical thrombectomies per annum. Until 2015, we primarily used a stent retriever technique, particularly with the Solitaire^TM^ FR stent. From 2016, we switched stepwise to the ADAPT technique using aspiration catheters.

Patients with internal carotid artery (ICA) occlusion or dissection who underwent additional ICA stenting as well as patients treated with both techniques or with another thrombectomy systems were excluded. We retrospectively evaluated and included 76 patients in this study; 21 patients were treated with the Solitaire^TM^ FR stent retriever system and 55 patients with the Penumbra ACE^TM^ aspiration catheter system. Due to the retrospective nature of the study and anonymized data processing, ethical board approval and informed patient consent was not mandatory according to local legislation (ethics committee of the Bavarian Medical Association). This study was performed in line with the principles of the Declaration of Helsinki of 1964 and its later amendments. The trial was registered at German Clinical Trials Register, ID: DRKS00023946.

Upon admission, the patients suspected to have AIS underwent a head computed tomography (CT) scan and CT angiography scan after the first neurological evaluation. Those with identified thrombotic proximal vessel occlusion without infarction demarcation and with National Institutes of Health Stroke Scale (NIHSS) [11] scores of >4 were selected for treatment with mechanical thrombectomy. In cases with no contraindications, a systemic bridging thrombolytic therapy with rtPA (0.9 mg/kg of body weight) was administered. The thrombectomy was performed under general anesthesia. A head CT scan was performed on day-one post-intervention. The efficacy outcome measurements were recanalization time (in minutes), defined as the time from groin puncture to maximal vessel recanalization; procedure time (in minutes), defined as the time from groin puncture to the end of the endovascular procedure; fluoroscopy time (in minutes), defined as the sum of x-ray time usage of both angiography planes; dose area product (in cGy.cm^2^); device passage count; and recanalization rate assessed by the thrombolysis in cerebral ischemia (TICI) [12] score. An unbiased senior radiologist, who was blinded to the type of thrombectomy system used, performed the radiographic analysis (ME). Pre-intervention CT angiography scans divided the patients into those with and those without pial arterial collateralization—defined as peripheral arterial contrast filling of the affected vessel territory. Post-intervention day-one head CT scans divided the patients into three groups: those with major infarction occupying more then 1/3 of the vessel territory; those with minor infarction occupying less than 1/3 of the vessel territory or basal ganglia; and those without any infarction. Depending on the intracerebral hemorrhage (ICH) volume shown by the day-one head CT scan, we defined groups as having major ICH occupying more than 1/3 of the vessel territory, having minor ICH occupying less than 1/3 of the vessel territory, and having no ICH. Clinical outcome measures included outcome evaluated by the NIHSS score at discharge; defined as a good outcome when NIHSS 0–4, and a poor outcome when NIHSS >4 or death. These measures also included NIHSS score improvement (NIHSS at admission compared to NIHSS at discharge) and duration of patient hospitalization in days. Radiographic outcome measurements showed incidence of major and minor ICH as well as major and minor infarction on day-one CT scans.

Statistical analysis was performed using SPSS version 22.0 (SPSS Inc., Chicago, IL, USA). For comparison of groups for differences in the means, the independent samples *t*-test was used for numeric variables, and the Mann–Whitney Rank Sum test was used for ordinal variables and for variables with non-normal data distribution. For nominal variables, the χ^2^-test and Fisher’s exact test, together with contingency tables, were used. Differences of mean and median values as well as differences of frequencies in the χ^2^-test and Fisher’s exact test were statistically significant if the *p* value was <0.05. The results were evaluated using mean values ± standard deviation (SD), medians with interquartile ranges (IQR), and frequency tables. In the multiple comparison testing of subgroups, an adjustment with Bonferroni correction was performed. The predictors of good clinical outcome were calculated using binary logistic regression, with NIHSS at discharge as a dependent variable; defined as a good outcome in cases of NIHSS 0–4, and a bad outcome when NIHSS >4. The variables entered into the binary logistic regression were divided into a treatment-dependent group and a treatment-independent group.

## 3. Results

### 3.1. Baseline Characteristics

A total of 76 patients with AIS who underwent mechanical thrombectomy were enrolled using the Solitaire^TM^ FR stent retriever system (n = 21, 27.6%) and the Penumbra ACE^TM^ aspiration catheter system (n = 55, 72.4%). Both groups were statistically comparable: mean ages were 64 ± 15 vs. 69 ± 17 years (*p* = 0.236); mean NIHSS scores on admission were 20 ± 9 vs. 19 ± 8 (*p* = 0.600); mean symptom onset to groin puncture time was 242 ± 108 min vs. 229 ± 155 min (*p* = 0.712); 71.4% of cases involved bridging intravenous rtPA administration in the Solitaire^TM^ FR group vs. 72.7% of cases in the Penumbra ACE^TM^ group (*p* = 0.910). Additionally, sex (*p* = 0.800), vessel territory (*p* = 0.563) and site (*p* = 0.832) were comparable (Table 1).

### 3.2. Clinical Outcomes

We observed no significant difference between NIHSS scores at discharge; the Solitaire^TM^ FR group had mean NIHSS 10 ± 13 and the Penumbra ACE^TM^ group had mean NIHSS 12 ±16 (*p* = 0.629). In total, 52.4% of cases in the Solitaire^TM^ FR group and 56.4% of cases in the Penumbra ACE^TM^ group had good clinical outcomes (NIHSS 0–4 at discharge), these results are statistically comparable within both thrombectomy systems (*p* = 0.756). Length of hospital stay remained comparable, with mean 13 ± 5 days in the Solitaire^TM^ FR group and mean 11 ± 6 days in the Penumbra ACE^TM^ group (*p* = 0.177). NIHSS improvement (NIHSS score at admission compared to NIHSS score at discharge) was also not significantly different (*p* = 0.415), with mean 10 ± 11 in the Solitaire^TM^ FR group vs. mean 7 ± 15 in the Penumbra ACE^TM^ group.

### 3.3. Efficacy Outcomes

Overall complete recanalization rate, defined as TICI 3, was achieved in 65 cases (85.5%); 14 cases in the Solitaire^TM^ FR group, and 51 cases in the Penumbra ACE^TM^ group (66.7% vs. 92.7%, *p* = 0.008). These TICI scores were reached by mean 3.1 ± 1.3 (median 3, IQR 2) device passage counts in the Solitaire^TM^ FR group vs. mean 1.5 ± 0.7 (median 1, IQR 1) device passage counts in the Penumbra ACE^TM^ group (*p* < 0.001). The recanalization times were mean 26 ± 15 min in the Penumbra ACE^TM^ group vs. mean 85 ± 37 min in the Solitaire^TM^ FR group. Additionally, the procedure times were mean 48 ± 23 min vs. 109 ± 39 min, and the fluoroscopy times were mean 15 ± 12 min vs. 38 ± 20 min. These statistics show that these times were, overall, significantly shorter for the Penumbra ACE^TM^ group (*p* < 0.001). Mean dose-area product was lower in the Penumbra ACE^TM^ group at 6806 ± 4049 cGy.cm2 vs. 10,775 ± 5848 cGy.cm2 in the Solitaire^TM^ FR group (*p* = 0.002) (Table 2).

### 3.4. Radiographic Outcomes

We observed no significant difference in frequency distribution (χ^2^ = 1.449, *p* = 0.485) between the Solitaire^TM^ FR and Penumbra ACE^TM^ groups with relation to infarct demarcation on the day-one head CT scan. In the subgroup analysis which utilized the Bonferroni correction, we found comparable major infarct demarcation (>1/3 of vessel territory) rates in 23.8% of cases in the Solitaire^TM^ FR group and 30.9% of cases in the Penumbra ACE^TM^ group (*p* = 0.587). Minor infarct demarcation (<1/3 of vessel territory) was observed in 47.6% of cases in the Solitaire^TM^ FR Group and in 32.7% of cases in the Penumbra ACE^TM^ group (*p* = 0.290). Concerning the volume of ICH shown by the day-one head CT scan, we did not find a significant difference in frequency distribution (χ^2^ = 5.441, *p* = 0.089) between the Solitaire^TM^ FR and Penumbra ACE^TM^ groups. In the subgroup analysis which utilized the Bonferroni correction, we found that the Penumbra ACE^TM^ group had significantly lower rates of major ICH; 5.5% of cases compared to 23.8% of cases in the Solitaire^TM^ FR group (*p* = 0.033). Incidence of minor ICH was comparable, with 4.8% of cases in the Solitaire^TM^ FR group and 5.5% of cases in the Penumbra ACE^TM^ group (*p* = 1.000) (Table 3).

### 3.5. Predictors of Clinical Outcome

The binary logistic regression confirmed the significance of the treatment-independent variables group model (χ^2^ = 23.87, *p* = 0.001, n = 76) and identified lower initial NIHSS scores (odds ratio (OR) 0.925, *p* = 0.031, all other things being equal) as a predictor of a good clinical outcome. The pial arterial collateralization shown on the admission CT angiography scan was the next predictor of a good clinical outcome (OR 7.236, *p* = 0.003, all other things being equal). Nagelkerke R Square was 0.36, this corresponds, according to Cohen´s effect size, to strong effect (Table 4).

In the treatment-dependent variables group the binary logistic regression model was also significant (χ^2^ = 40.94, *p* < 0.001, n = 76); it identified a recanalization time of up to 40 min (OR 12.569, *p* = 0.02, all other things being equal) and the count of device passage (OR 2.523, *p* = 0.04, all other things being equal) as good clinical outcome predictors. In cases of major infarction (>1/3 of vessel territory) on day-one head CT scans, a good clinical outcome was 30 times less likely (OR 0.033, *p* < 0.001, all other things being equal). Nagelkerke R Square was 0.55, this corresponds, according to Cohen´s effect size, to strong effect (Table 5).

## 4. Discussion

This study compared the technique of mechanical thrombectomy with stent retriever, using the Solitaire^TM^ FR Stent retriever system, with the direct thrombus aspiration technique, using the Penumbra ACE^TM^ aspiration catheter system. In the Penumbra ACE^TM^ cohort we observed better TICI 3 recanalization rates, lower device passage counts, shorter procedure and recanalization times, and lower radiation loads. Clinical outcomes were statistically comparable between both cohorts.

Baseline characteristics were comparable between both groups. Mean age in our population correlates with the data in the literature [8,13,14], but the admission NIHSS scores and onset of symptoms to groin puncture times differ between the trials. Higher incidence of medial cerebral artery occlusion also corresponds to data in the literature, but the incidence of basilar artery occlusion was higher in our population, with 14.5% vs. 10% [14]. The frequency of intravenous rtPA administration prior to intervention was lower in our population, with 72.4% of cases compared to the published 89% [2].

The clinical outcomes, as evaluated by NIHSS scores at discharge, length of hospitalization, and NIHSS improvement, were statistically comparable within both thrombectomy systems. We report a good clinical outcome in 52.4% of patients in the Solitaire^TM^ FR group and in 56.4% of the Penumbra ACE^TM^ group. Comparable clinical outcomes between ADAPT and stent retriever groups correlate with the findings of Stapleton et al. [13], Lapergue et al. [8], Turk et al. [9] and Primiani et al. [15].

In accordance with other trials [15,16], we observed higher rates of complete vessel recanalization (TICI 3) in the Penumbra ACE^TM^ aspiration group. The lower recanalization rate in the Solitaire^TM^ FR stent retriever group could be affected by the smaller cohort or possibly by the lower initial experience of the interventionists. When comparing our results to the results of multicenter randomized trials, published in 2015, that reported recanalization rates ranging between 59–88% [2,3,4,5,6], we achieved average recanalization rate results in the Solitaire^TM^ FR stent retriever group. The materials and experience of the interventionists are developing over the years. Nevertheless, the results of a multicenter randomized trial comparing the stent retriever technique and the ADAPT technique, published in 2019, showed mean recanalization rates of 68.9% in the stent retriever group [9] which were similar to our findings. Additionally, another trial, published in 2021, comparing three large-bore aspiration catheters found no differences in terms of successful recanalization [17]. Phan et al. reported that a direct contact thrombus aspiration as a first pass technique reduces the recanalization time. The crux of the ADAPT technique is its philosophy in starting with the simple step of aspiration with an atraumatic and easily tractable catheter. This method can be escalated to the more complex use of stent retrievers if the aspiration was not successful [16]. Concerning our data evaluating technical efficacy, we found shorter recanalization times, shorter fluoroscopy times, lower dose area products, shorter overall procedure times and lower device passage counts in the Penumbra ACE^TM^ aspiration group. In our opinion, the use of aspiration catheters could be advantageous in cases of proximal large-vessel occlusion where the manipulation with a large-bored catheter allows for a more straightforward procedure. Primarily, usage of the stent retriever technique could be better for more complex anatomical conditions, more peripheral localized vessel occlusion, and in cases of the failure of the aspiration technique. However, there is currently very limited evidence that evaluates the role of vascular anatomy on the outcomes of the ADAPT strategy [16]. According to our findings, these factors allow for the usage of the direct aspiration technique which is a quicker and a more effective way to achieve maximal vessel recanalization and which reduces the radiation load on the patient and the medical staff, compared to the stent retriever technique. Due to the lack of data in the literature these findings have to be proven by future studies. We report no differences between the groups in relation to major infarct demarcations on the 24 h CT scan. However, a higher rate of major intracerebral hemorrhage (*p* = 0.033) was observed in the Solitaire^TM^ FR stent retriever group; 23.8% vs. 5.5% of cases. Primiani et al. [15] also reports a higher rate of ICH in stent retriever group with 7.2% vs. 5.6%. However, Turk et al. [9] reports 36% vs. 34%, with the higher rates of ICH in the aspiration group. Based on the literature the incidence of infarct demarcation and bleeding complications differ between the trials. Salsano et al. analyzed the complications of endovascular thrombectomy for large-vessel occlusion (LVO) strokes and found that a higher NIHSS score at onset, longer groin-to-reperfusion time and the site of the LVO (carotid T as well as M2 MCA) were associated with a higher risk of developing symptomatic ICH compared to no/asymptomatic ICH [18].

Regarding the predictors of clinical outcome, we identified in the group of treatment-dependent variables that a recanalization time of up to 40 min and the count of device passages were predictors of good clinical outcomes. These findings could imply that quicker vessel recanalization is associated with better prognosis for the treated patient. As expected, major infarct demarcation shown on the 24 h CT scan was associated with a lower chance for a good clinical outcome. In accordance with the findings of Barral et al. [19], Gamba et al. [20], Lu et al. [21] and Alexandre et al. [22] the lower initial NIHSS scores in our study also favor a good clinical outcome. In relation to clinical results of the treatment in posterior circulation strokes, the lower baseline NIHSS score and the thrombectomy using large-bore aspiration catheters were predictors of a good clinical outcome [23]. Pial arterial collateralization in the admission CT angiography scan was another strong predictor of good clinical outcome. According to our findings, as well as the reports of Liebeskind et al. [24], Woo et al. [25] and Christoforidis et al. [26], good pial arterial collaterals on the initial CT angiography scan, in cases of the successful vessel recanalization, can be crucial for a good clinical outcome. Rabinstein reports that the collateral status and the time from onset of symptoms to vessel reperfusion are the main determinants of the clinical outcome [27,28]. In our study, the time from symptom onset to groin puncture had no predictive value regarding a good clinical outcome. Alexandre et al. reviewed the data of patients treated for LVO later than six hours after symptom onset and found that a perfusion CT scan can provide better patient selection compared to a CT angiography alone [22]. In our opinion, the evaluation of pial arterial collateralization on initial CT angiography scans can contribute to outcome estimation without the need for an additional perfusion CT scan or an MRI. Furthermore, the presence of pial arterial collaterals on initial CT angiography, could, without the need for additional time-consuming investigations, contribute to an indication for treatment with mechanical thrombectomy in patients with wake-up stroke. The use of multiphase CT angiography allows for better localization of arterial occlusion and provides a spatial and temporal evaluation of the patency and status of the collateral circulation. Usage of the summated time variant color-code map (ColorViz) allows an even more immediate and clearer visual impact, and avoids the need to compare three different phases at the same time, thus resulting in faster and more understandable diagnostic evaluation for stroke clinicians [29].

In this study the primary target was to compare the procedural and technical factors of the stent retriever technique, with Solitaire^TM^ FR, and the direct aspiration technique, with Penumbra ACE^TM^ aspiration catheter. In contrast to the comparison of clinical outcomes, these aspects have not yet been well described in the literature.

We acknowledge several limits of this clinical study. Firstly, the retrospective nature of the research and the corresponding biases that can be associated with this study design. Secondly, the monocentric character of the study. The next limitation was the relatively small cohort of included patients. Nevertheless, we think that we have depicted the representative population.

## 5. Conclusions

Mechanical thrombectomy in stroke due to LVO using the Penumbra ACE^TM^ aspiration catheter and the Solitaire^TM^ stent retriever system result in comparable clinical outcomes. Usage of aspiration catheters could be advantageous in cases of proximal large-vessel occlusion where the manipulation with a large-bored catheter is possible and allows for a more straightforward procedure. On the other hand, the usage of the stent retriever allows for the treatment of more challenging anatomical conditions and cases of more peripheral localized vessel occlusion. Good pial arterial collaterals on the initial CT angiography scan, lower baseline NIHSS scores, as well as quick vessel recanalization, are predictors of a good clinical outcome.

## Figures and Tables

**Table 1:** Baseline characteristics of Solitaire^TM^ FR stent retriever group and Penumbra ACE^TM^ aspiration catheter group.

		Solitaire^TM^ FR	Penumbra ACE^TM^	*p*-Value
		n = 21, 27.6%	n = 55, 72.4%	
Mean age in years (SD)		64 (15)	69 (17)	0.236 *
NIHSS scores on admission (SD)		20 (9)	19 (8)	0.600 *
Symptom onset to groin puncture time in minutes (SD)		242 (108)	229(155)	0.712 *
Female sex (%)		9 (42.9%)	26 (47.3%)	0.800
Intravenous rtPA administration (%)		15 (71.4%)	40 (72.7%)	0.910
Vessel territory (%)	BA	4 (19.0%)	7 (12.7%)	0.563
	ICA	3 (14.3%)	13 (23.6%)	
	MCA	14 (66.7%)	35 (63.6%)	
Site (%)	left	9 (42.9%)	24 (43.6%)	0.832
	right	8 (38.1%)	24 (43.6%)	
	posterior	4 (19.0%)	7 (12.7%)	

For the marked values (*) the independent samples *t*-test was used. For other cases, when the χ^2^-test and Fisher’s exact test were used, a 2-sided *p*-value is presented. BA = Basilar artery; ICA = Internal carotid artery; MCA = Middle cerebral artery; NIHSS = National Institutes of Health Stroke Scale; rtPA = Recombinant tissue plasminogen activator; SD = Standard deviation.

**Table 2:** Efficacy outcomes in the Solitaire^TM^ FR stent retriever group and the Penumbra ACE^TM^ aspiration catheter group.

		Solitaire^TM^ FR	Penumbra ACE^TM^	*p*-Value
		n = 21, 27.6%	n = 55, 72.4%	
TICI recanalization score (%)	3	14 (66.7%)	51 (92.7%)	0.008 *
	2b	1 (4.8%)	2 (3.6%)	
	2a	4 (19%)	2 (3.6%)	
	1	2 (9.5%)	0 (0.0%)	
Mean recanalization time in minutes (SD)		85 (37)	26 (15)	0.000
Mean procedure time in minutes (SD)		109 (39)	48 (23)	0.000
Mean fluoroscopy time in minutes (SD)		38 (20)	15 (12)	0.000
Mean dose area product in cGy.cm^2^		10,775 (5848)	6806 (4049)	0.002
Mean device passage count		3.1 (1.3)	1.5 (0.7)	0.000

For the marked values (*) the χ^2^-test was used. For other cases, when the independent samples *t*-test was used, a 2-sided *p*-value is presented. SD = Standard deviation; TICI = Thrombolysis in cerebral ischemia.

**Table 3:** Radiographic outcomes in the Solitaire^TM^ FR stent retriever group and the Penumbra ACE^TM^ aspiration catheter group.

		Solitaire^TM^ FR	Penumbra ACE^TM^	*p*-Value
		n = 21, 27.6%	n = 55, 72.4%	
Infarct on day-one CT scan (%)	>1/3 of territory	5 (23.8%)	17 (30.9%)	0.587
	<1/3 of territory	10 (47.6%)	18 (32.7%)	0.290
	No infarct	6 (28.6%)	20 (36.4%)	0.597
ICH on day-one CT scan (%)	>1/3 of territory	**5** (**23.8%**)	**3** (**5.5%**)	**0.033**
	<1/3 of territory	1 (4.8%)	3 (5.5%)	1.000
	No ICH	15 (71.4%)	49 (89.1%)	0.080

For calculation of frequencies the χ^2^-test and Fisher´s exact test were used, 2-sided *p*-values are presented. Tests are adjusted for all pairwise comparisons within a row of each innermost subtable, using the Bonferroni correction. Values marked in bold are significantly different at *p* < 0.05. ICH = Intracerebral hemorrhage.

**Table 4:** Predictors of good clinical outcome (NIHSS 0–4) calculated using binary logistic regression. Treatment-independent group of variables.

Treatment-Independent	OR (95% CI for OR)	*p*-Value
Variables		
Age	0.969 (0.934–1.005)	0.091
Sex	0.552 (0.169–1.797)	0.324
Symptom onset to groin puncture time	0.998 (0.995–1.002)	0.409
Intravenous rtPA administration	1.215 (0.361–4.084)	0.753
Pial arterial collateral supply	**7.236** (**1.956–26.760**)	**0.003**
Initial NIHSS	**0.925** (**0.862–0.993**)	**0.031**

Dependent variable NIHSS at discharge (good outcome NIHSS 0–4) entering the binary logistic regression model. Values marked in bold are significantly different at *p* < 0.05. CI = Confidence interval; NIHSS = National Institutes of Health Stroke Scale; OR = Odds Ratio.

**Table 5:** Predictors of good clinical outcome (NIHSS 0–4) calculated using binary logistic regression. Treatment-dependent group of variables.

Treatment-Dependent	OR (95% CI for OR)	*p*-Value
Variables		
**Recanalization time up to 40 min**	**12.659** (**1.489–107.639**)	**0.020**
Thrombectomy system	2.002 (0.157–25.580)	0.593
TICI 1 recanalization	0.000 (0.000)	0.999
TICI 2a recanalization	0.757 (0.007–82.781)	0.908
TICI 2b recanalization	6.881 (0.007–6641.137)	0.582
Device passage count	**2.523** (**1.025–6.214**)	**0.044**
Infarction >1/3 of territory	**0.033** (**0.005–0.215**)	**0.000**
Infarction <1/3 of territory	0.272 (0.059–1.263)	0.097
ICH >1/3 of territory	0.164 (0.010–2.586)	0.199
ICH <1/3 of territory	0.133 (0.006–2.962)	0.202

Dependent variable NIHSS at discharge (good outcome NIHSS 0–4) entering the binary logistic regression model. Values marked in bold are significantly different at *p* < 0.05. CI = Confidence interval; ICH = Intracerebral hemorrhage; TICI = Thrombolysis in cerebral ischemia; OR = Odds Ratio.

## Data Availability

The data presented in this study are available on request from the corresponding author.
